# Editorial: Molecular mechanisms in neural development, related disorders, and therapeutic treatments

**DOI:** 10.3389/fnmol.2023.1135491

**Published:** 2023-01-13

**Authors:** Vijay Kumar, Kausik Bishayee, Jaebong Kim

**Affiliations:** ^1^Department of Biochemistry, College of Medicine, Institute of Cell Differentiation and Aging, Hallym University, Chuncheon, South Korea; ^2^Biomedical Science Core-Facility, Soonchunhyang Institute of Medi-Bio Science, Soonchunhyang University, Cheonan-si, South Korea

**Keywords:** neurological disease, signaling, genetic factors, diagnosis, therapeutic treatment

Despite the availability of treatments or modern prevention methods, neurological diseases are the leading cause of death and a considerable global concern, affecting overall human health (Chin and Vora, 2014). Neurological research requires sustained efforts to investigate disease etiology and pathogenesis and develop diagnostic and prevention strategies. Increasing evidence suggests that neural pathogenesis is associated with compromised molecular events driving neuronal development, shaping, and functionality, which may occur at any stage of life. Neurons exhibit limited regenerative capacity, which demands a deep understanding of the molecular mechanisms underlying the pathogenic causes, including genetic factors, flawed molecules, signaling pathways, and loss/gain-of enzyme activity. Recently, remarkable progress has been made in identifying the target molecules and their association with pathophysiological processes. This Research Topic aims to analyze the molecular events, associated biomolecules, signaling pathways, and mechanisms underlying neurological diseases and their applicability as potential therapeutic targets.

Among the several types of brain tumors, glioma is the most common intra-axial tumor originating from glial cells and accounts for 33 % of all the brain tumors (Hanif et al., 2017). In addition, a high recurrence and mortality rate results in only 5.5 % of the patients surviving after diagnosis for a median 14.5 to 16.6 months. Several key signaling pathways, including Hippo/YAP, Notch, Pi3K/AKT/mTOR, TGF-β, and Wnt/β-catenin, play essential roles in normal biological processes such as proliferation, differentiation, and migration. However, an irregularity in any of these signaling pathways promotes tumorigenesis and neurological disorders (Wu et al.). Wu et al. summarized the mechanisms associated with glioma pathogenesis and alterations in signals involved in glioma progression and malignancy. Based on existing evidence, they explored the possibility of targeting key molecules or pathways, such as Notch, Yap1, and miRNA, which may help develop treatment and diagnosis strategies (Wu et al.).

WNT signaling plays an essential role in neuronal development and functionality. However, impaired Wnt signaling is involved in tumorigenesis (particularly gliomagenesis). Similarly, Alkailani et al. reviewed research evidence representing the active involvement of WNT signaling in brain tumorigenesis. This study primarily focused on the mechanism of WNT signaling contributing to glioblastoma (GMB) progression. For example, Wnt degradation/dysregulation in GMB progression and activation of Wnt signaling by oncogenes, such as WNTless (WLS/Gpr177), have been reported in WNT ligand secretion (Alkailani et al.).

Autophagy is an essential cellular process implicated in various pathophysiological conditions, including cancer and neurodegenerative disorders. It is characterized by the sequestration of cellular cargo (autophagosome), which fuses with lysosomes to degrade harmful molecules (Meng et al., 2019). Due to enlarged dendritic and axonal cytoplasm, autophagic vacuoles (AV) travel considerable distances to fuse with lysosomes. Thus, the hindering of organelle transport in neurons causes the accumulation of AV and other waste over time, which promotes neurodegenerative diseases such as Alzheimer's disease (Lee et al., 2011). WD-repeat protein interacting with phosphoinositides (WIPI) binds to phosphoinositides and recruits autophagy proteins, thus playing an important role in autophagy. Almannai et al. has summarized the biological functions of WIPI proteins (WIPI1, WIPI2, WIPI3, and WIPI4) in human diseases. Neural tube defects in a patient cohort study and convergent extension defects in WIPI1 knockdown/overexpression zebrafish indicate the crucial role of WIPI1 in neural tube formation. However, based on transcriptomic data, high WIPI1 signaling contributes to right ventricular failure (Almannai et al.). Phosphorylated WIPI2 is involved in autophagosome biogenesis. In aged mice, phosphorylation misregulation triggers autophagic structure accumulation and a decline in the neuronal autophagy process. In addition, defective *WIPI2* induces several abnormalities such as intellectual disability, reduced brain volume, microcephaly, seizures, skeletal-related abnormalities, and dysmorphic features. WIPI3 is involved in the pathogenesis of El-Hattab-Alkuraya syndrome, with profound global developmental delay, microcephaly, early onset refractory seizures, and progressive spastic quadriplegia (Almannai et al.). Furthermore, WIPI4 is associated with the pathogenesis of Beta-propeller protein-associated neurodegeneration. Therefore, the normal expression of WIPI proteins is necessary for the impaired expression of normal cellular states driving pathophysiological conditions (Almannai et al.).

Spinocerebellar Ataxia (SCA) is an inherited neurodegenerative disorder affecting the brain cerebellum (sometimes the spinal cord) and regulates physical coordination and movement. Approximately 40 SCA types been genetically identified; among these, SCA3, accounting for approximately 35 % of the SCAs, is the most preventable form (Seidel et al., 2012; Diallo et al., 2021). Li et al. The c.1130 C>T (p.T377M) mutation in the *KCND3* gene (encoding potassium voltage-gated channel subfamily D member 3 (Kv4. 3) protein), which induces protein misfolding and aggregation, and further endoplasmic reticulum stress collectively leads to neuronal apoptosis in SCA19/22.

## Closing remarks

Although there is increased interest in neurodegenerative diseases recently, its molecular mechanisms and pathogenic causes are still unclear. Understanding molecular factors and signaling pathways can aid in developing accurate prognosis, diagnosis, and therapeutic interventions, which are essential for treating neurodegenerative diseases.

## Author contributions

VK wrote the original draft. KB helped with revising of the manuscript. JK supervised the study. All authors have read and approved the published version of the manuscript.
